# Phylogenetic Characteristics of Anthrax Outbreaks in Liaoning Province, China, 2001-2015

**DOI:** 10.1371/journal.pone.0157496

**Published:** 2016-06-14

**Authors:** Lingling Mao, Enmin Zhang, Zijiang Wang, Yan Li, Hang Zhou, Xuesheng Liu, Huijuan Zhang, Hong Cai, Xudong Liang, Yingwei Sun, Zhikai Zhang, Wei Li, Wenqing Yao, Jianchun Wei

**Affiliations:** 1 Liaoning Provincial Center for Disease Control and Prevention, Shenyang, Liaoning Province, China; 2 National Institute for Communicable Disease Control and Prevention, China CDC, Changping, Beijing, China; 3 State Key Laboratory for Infectious Disease Prevention and Control, Beijing, China; 4 Collaborative Innovation Center for Diagnosis and Treatment of Infectious Disease, Hangzhou, China; 5 Tieling Prefecture Center for Disease Control and Prevention, Tieling, Liaoning Province, China; 6 Division of Infectious Disease Prevention and Control, China CDC, Beijing, China; Centers for Disease Control and Prevention, UNITED STATES

## Abstract

Anthrax is a continuous threat in China, especially in rural regions. In July 2015, an anthrax outbreak occurred in Xifeng County, Liaoning Province. A total of 10 cutaneous anthrax cases were reported, with 210 people under medical observation. In this study, the general characteristics of human anthrax outbreak occurred in Liaoning Province were described, and all cases were caused by butchering and contacting sick animal. Meanwhile, the phylogenetic relationship between outbreak-related isolates/samples of the year 2015 and previous *Bacillus anthracis* strains was analyzed by means of canonical single nucleotide polymorphisms (canSNP), multiple-locus variable-number tandem repeat analysis (MLVA) with 15 markers and single-nucleotide repeats (SNR) analysis. There are two canSNP subgroups found in Liaoning, A.Br.001/002 and A.Br.Ames, and a total of six MLVA 15 genotypes and five SNR genotypes were observed. The strain collected from anthrax outbreak in Xifeng County in 2015 was classified as A.Br.001/002 subgroup and identified as MLVA15-29 genotype, with same SNR profile (CL10: 17, CL12: 15, CL33: 29, and CL35: 13). So we conclude that the same clone of *B*.*anthracis* caused the anthrax outbreak in Xifeng County in 2015, and this clone is different to previous isolates. Strengthening public health education in China is one of the most important measures to prevent and control anthrax.

## Introduction

Anthrax is an acute zoonotic infectious disease caused by *Bacillus anthracis* (*B*. *anthracis*). *B*. *anthracis* can form dormant spores that are able to survive in harsh conditions and persist for long periods in soil. Herbivores and domestic livestock, the natural hosts of *B*.*anthracis*, are usually infected by contact with spore-containing soil [1]. There are three primary forms of anthrax in humans including inhalation, gastrointestinal, and cutaneous anthrax. Cutaneous anthrax is the most common form. Cutaneous anthrax infection in humans is acquired from close contact with infected animals or contaminated animal products, such as meat and skin etc. Anthrax has been reported from most countries of the world [1]. *B*.*anthracis* spores have also been considered a potential biological weapon [2]. One of the most notorious bioterrorism events related to *B*. *anthracis* was “letter attacks” in the United States in 2001 [3].

*B*.*anthracis* is a genetically homogeneous pathogen [4]. Investigation of the bioterrorism-associated anthrax attack in 2001 and other anthrax outbreaks promoted the development of molecular subtyping of *B*.*anthracis* isolates, such as single nucleotide polymorphism (SNP), multiple-locus variable-number tandem repeat analysis (MLVA), etc[5]. SNP analysis has been used to illustrate the phylogenetic relationship and genomic evolution of *B*.*anthracis* isolates [6,7,8,9]. Pearson et al. demonstrated an extremely conserved clonal population structure for this species by genome-wide SNP analysis [6]. A genotyping method that uses a small number of canonical SNPs (canSNP) to replace the genome-wide SNP analysis has been developed and used to identify a broad genetic group of *B*. *anthracis* [5]. Variable-number tandem repeats (VNTRs) were successfully developed as molecular markers by Keim et al., and its discriminatory power is greatly enhanced if multiple loci are examined concurrently. MLVA genotyping systems have also been developed by different teams [10, 11, 12]. Single-nucleotide repeats (SNRs) are a class of VNTRs that display very high mutation rates. SNRs were found to provide additional genetic resolution among B. anthracis strains with the same MLVA genotype [5, 13]

Since these methods have different resolution power, Keim et al. has suggested that a combination of VNTRs and SNPs represents a better approach, termed PHRANA (Progressive hierarchical resolving assays using nucleic acids) [5]. PHRANA is a nested hierarchal approach that employs canSNPs, MLVA-15 and, finally, the SNR-4 system to accurately characterize phylogenetic relationships among *B*.*anthracis* isolates. The global genetic population structure of *B*.*anthracis* had also been defined by the canSNP and MLVA methods subsequently [4]. MLVA has considerable resolution power in subtyping *B*.*anthracis*, and had been used in investigation of bioterrorism attack associated with *B*. *anthracis* spores [3]. The canSNP and MLVA methods have also been wildly used to illustrate the phylogenetic relationship of *B*.*anthracis* in different countries and as source tracing methods in the anthrax outbreaks [14, 15, 16, 17].

Anthrax is an endemic disease and breaks out every year in China, with occasional deaths, especially in rural regions of the western areas of China. Anthrax outbreaks frequently occurred in livestock and humans in Liaoning Province. From national epidemiological surveillance data, we observed that Liaoning Province suffered human anthrax heavily before 1980s, such as 408 cases were reported in 1950s, 250 cases in 1960s, and 242 cases in 1970s. After 1980s, with the development of economy the number of human anthrax decreased greatly. However, the total number of human cases remained high, such as 33 cases in 1980, 42 cases in 1990. A total of 109 human anthrax cases were reported in Liaoning between 2001 and 2015. The most serious outbreak occurred in Anshan prefecture in 2011, with at least 34 human cases reported and 456 farm livestock dead or slaughtered in suffered areas [18]. At the beginning of August in 2015, one outbreak with 10 suspected cutaneous anthrax cases was reported in Xifeng County, Liaoning province, with a total of 210 people under medical observation. In this study, we reported the outbreak and investigated the phylogenetic relationship between the outbreak-related isolate/samples and these *B*.*anthracis* strains isolated before.

## Materials and Methods

### Ethics Statement

All procedures performed in the study were in accordance with the ethical standards of the institutional and national research committee. The body fluid samples were collected from blister fluid of the patients’ skin lesions after obtaining the patients’ oral informed consent. The consents were documented by the attending physician in the local hospital of Liaoning Province. The oral informed consents and the study were approved by the Review Board of Ethics in National Institute for Communicable Disease Control and Prevention, China CDC (Permit number: ICDC-2014008).

### Strains and samples

One strain isolated from dead cattle, 10 blister fluid samples from suspected anthrax patients and 3 raw bone or skin samples from dead cattle in Xifeng County, Liaoning Province, 2015, were analyzed (Table 1). A total of 7 *B*.*anthracis* strains collected during 2001–2014 in Liaoning Province were also included in this study, including 4 strains associated with human anthrax cases occurred in Liaoning in 2007 and 2011. In addition, 21 *B*.*anthracis* strains collected from other provinces were also included in this study. The VNTRs profiles and canSNP data of 30 *B*.*anthracis* strains collected in China (15 of them from Liaoning province) and 12 international strains in previously published study were also included into the analysis [18].

**Table 1:** *B*. *anthracis* isolates used in this study and the human anthrax occurred in Liaoning in 2001–2015.

Year	Strain (Source)	Province	Prefecture (number of patient)
	Isolates from Liaoning		
**2001**	No	Liaoning	Shenyang(1),Jinzhou(29), Fuxin(1),Tieling(1)
**2002**	LN 149(soil)	Liaoning	0
**2003**	LN 150(soil), LN 151(soil)	Liaoning	0
**2005**	No	Liaoning	Shenyang(11)
**2007**	LN152(chopping board)	Liaoning	Tieling(4)
**2008**	No	Liaoning	Shenyang(2)
**2011**	HC2001(Patient),LN153(Patient), LN154(Patient)	Liaoning	Shenyang(1),Anshan(29), Dandong(4)
**2012**	No	Liaoning	Shenyang(4),Anshan(1), Jinzhou(2)
**2013**	No	Liaoning	Dandong(1)
**2014**	No	Liaoning	Shenyang(4),
**2015**	^a^T1-10(Patient,10^b^), strain T11 (cattle), ^a^T12-14(bone and skin of cattle,3^b^)	Liaoning	Tieling(10), Shenyang(2), Liaoyang(2)
	Isolates from other provinces		
**1953**	GD85(Buffalo)	Guangdong	Unknown
**1955**	NM35(Bovine)	Inner mongolia	Unknown
**1993**	GX62(soil)	Guangxi	Unknown
**1994**	GX70(soil, 2^b^)	Guangxi	Unknown
**1994**	GX60(patient)	Guangxi	Unknown
**1995**	GX66(patient)	Guangxi	Unknown
**1995**	XJ12(sheep)	Xinjiang	Unknown
**1995**	XJ17(Unknown)	Xinjiang	Unknown
**1997**	NM75(Bovine)	Inner mongolia	Unknown
**1997**	NM76(sheep)	Inner mongolia	Unknown
**1998**	NM72(soil, 2^b^)	Inner mongolia	Unknown
**1998**	NM74(patient)	Inner mongolia	Unknown
**2005**	QH115(patient)	Qinghai	Unknown
**2006**	SC103(patient)	Sichuan	Unknown
**2011**	NM122(patient, 4^b^)	Inner mongolia	Unknown
**2012**	NM130(patient)	Inner mongolia	Unknown
	Isolates from literature		
**1952**	A0560CHI(soil, 14^b^)	Liaoning	Unknown
**1952**	A0561CHI(soil, 1^b^)	Liaoning	Unknown
**1956**	A0552CHI(patient, 5^b^)	Hebei	Unknown
**1962**	A0581CHI(patient, 2^b^)	Inner Mongolia	Unknown
**1962**	A0582CHI(patient)	Inner Mongolia	Unknown
**1962**	A0583CHI(patient)	Inner Mongolia	Unknown
**1973**	A0590CHI(Unknown)	Gansu	Unknown
**1982**	A0592CHI(pig)	Gansu	Unknown
**1958**	A0594CHI(patient)	Gansu	Unknown
**unknown**	A0595CHI(Unknown)	Sichuan	Unknown
**1957**	A0721CHI(Unknown)	Unknown	Unknown
**1957**	A0729CHI(Unknown)	Unknown	Unknown
**Unknown**	A0077AUS(Unknown)	Unknown	Unknown
**Unknown**	A0196CAN(Unknown)	Unknown	Unknown
**Unknown**	A0330GER(Unknown)	Unknown	Unknown
**Unknown**	A0332GER(Unknown)	Unknown	Unknown
**Unknown**	A0341GER(Unknown)	Unknown	Unknown
**Unknown**	A0394USA(Unknown)	Ames	Unknown
**Unknown**	A0935THA(Unknown)	#1 Tak Province	Unknown
**Unknown**	A0936THA(Unknown)	#2 Tak Province	Unknown
**Unknown**	A0937THA(Unknown)	Lopburi Province	Unknown
**Unknown**	A1045USA(Unknown)	Unknown	Unknown
**Unknown**	A1115USA(Unknown)	Unknown	Unknown
**Unknown**	A2012USA(Unknown)	Unknown	Unknown
**Total**	71(strains),13(samples)		109 patients in Liaoning

^a^ Samples.

^b^ Number of the strains with same information.

### DNA preparation and real-time PCR assays

*B*. *anthracis* strains were streaked onto LB agar plates and incubated at 37°C for 16–18 h. A single colony was suspended in 0.5 ml of TE buffer (10.0mM Tris-HCl [pH 8.0], 1.0 mM EDTA) and incubated at 100°C for 20 min. Then, cellular debris was removed by centrifugation at 15,000×g for 10 min. The supernatant was collected and filtered using 0.22μm filters. The filtered supernatant was diluted 1:10 with sterile nuclease-free H_2_O and used as DNA template for PCR amplification. The bacterial culture and preparation of DNA templates were performed in Bio-Safety level 3 (BSL3) Laboratory. Blister fluid specimens collected from patients were used to extract genomic DNA according to DNeasy Blood & Tissue Kit (QIAGEN) manual. These DNA templates were screened by Anthrax real-time PCR Kit (Shanghai Huirui Biotechnology Co., Ltd), including 10μl 2× reaction mix, 0.2 μM each pairs of primers and TaqMan probes targeting the protective antigen gene (*pag*A), capsule synthesis gene (*cap*C) and chromosomal *rpoB* genes, 5 μl DNA template and sterile nuclease-free H_2_O to a final volume of 20 μl.

### Molecular genotyping

The canSNP analysis with 13 markers was performed as described by Van Ert et al [4]. The MLVA15 analysis was performed as described previously [4, 19] with the following modifications on capillary electrophoresis. The forward primers were labeled with different fluorescent dyes (Fam, Hex). PCR amplifications were performed on SensoQuest Labcycler with a starting denaturation step at 95°C for 5 min, followed by 34 cycles of denaturation at 95°C for 30 s, annealing at 60°C for 30 s (55°C for vrrB1 and vrrB2, 52°C for VNTR12, VNTR16 and VNTR17)and extension at 72°C for 1 min. The reactions were terminated by a final incubation at 72°C for 5 min. The amplicons were diluted in water to 1:100 (for DNA templates from patients’ blister fluid sample, no diluted for amplicons), after denaturation by heating, the amplicons were separated by capillary electrophoresis on an ABI 3730xl genetic analyzer with a GeneScan 1200 LIZ size standard (Applied Biosystems). The lengths of amplicons were determined according to size using GeneMapper software V. 4.0(Applied Biosystems). The nomenclature of MLVA15 genotypes described by Van Ert et al [4] was used. The SNR analysis with 4 markers was performed as described by Kenefic et al [20]. To determine the number of single-nucleotide repeats, some PCR amplicons were sequenced with the primers described by Beyer et al [21].

### Cluster analysis of data from canSNP, MLVA and SNR

Data were imported into the BioNumerics software (version 5.10, Applied-Maths) as character data sets. Data from canSNP, MLVA and SNR analysis were processed by Clustering analysis using the categorical coefficient and the unweighted pair-group method with arithmetic means. Cluster analysis of the categorical data was presented using dendrograms.

## Results and Discussion

### Anthrax outbreak in Xifeng County, Liaoning, 2015

**Fig 1:**
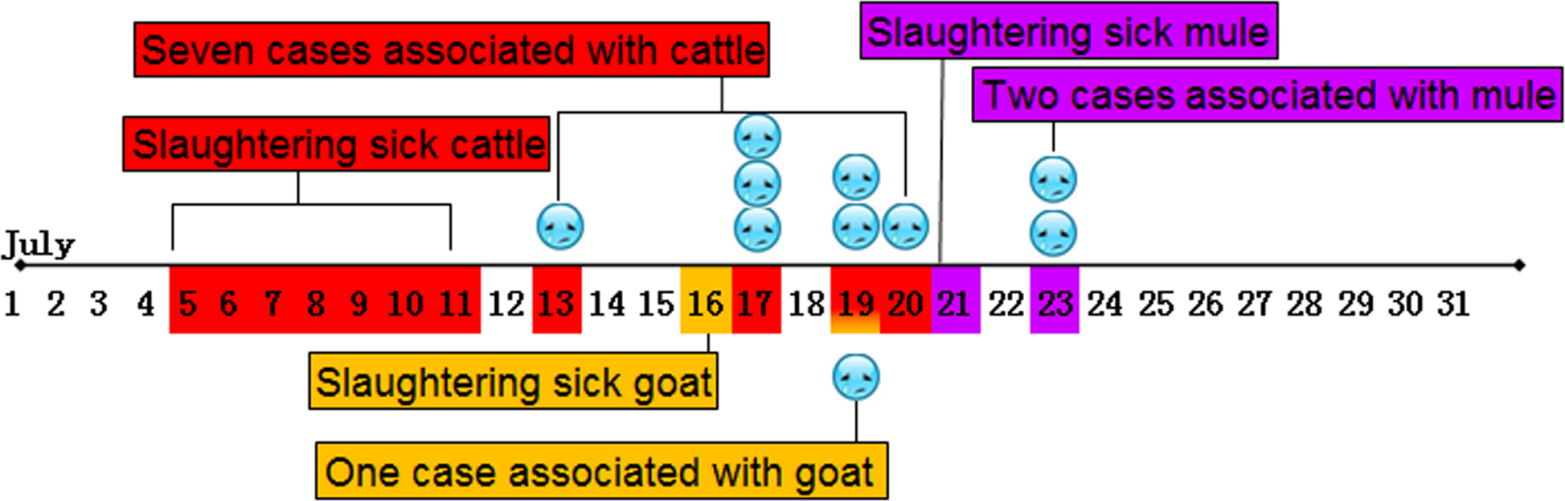
Exposure and onset time of the 10 confirmed cases in Xifeng County, Liaoning Province. There are three colors represent the exposure and onset time of the 10 confirmed cases from three events. The event in red: from July 5 to 11, sick cattle were slaughtered and there were 7 cases associated with the event. The event in orange: slaughtering sick goat happened on July 16, and one case appeared on July 19. The event in purple: a sick mule was slaughtered on July 21, and that caused 2 cases on July 23.

On August 5 in 2015, an outbreak with 10 suspected cutaneous anthrax human cases (one female, nine males) was reported by the health authorities of Xifeng County, Liaoning Province. These patients were scattered in seven villages and associated with three exposure events. The first event involved butchering 17 sick cattle in a farm from July 5 to 11, where a total of ten farmers participated in slaughtering or skinning, and six were infected, including the index patient (60 years old, male) who suffered cutaneous anthrax on fingers on July 13 (Fig 1). In addition, one case (female) contacted raw meat of sick animal on July 11 and got infection. The second event caused one cutaneous anthrax case who slaughtered a dead goat. The third event suffered two cutaneous anthrax cases who butchered a sick mule on July 21. Both of the latter two events happened around the cattle farm. Through an expanded search in the affected villages, two more people were identified as suspected cases. A total of 210 people who contacted infected animals or contaminated animal products were under medical observation, and no anthrax cases were reported among these people during the observation period. Together with epidemiologic, clinical, and laboratory data, a total of 10 confirmed patients were identified according to the criteria of World Health Organization [1].

There were more than 1100 livestock in affected villages, where livestock were rarely vaccinated. The affected farm once brought 120 cattle from Dalian (about 600km away from the suffered county) on July 3 in 2015, and these cattle were not quarantined and directly melt into herds on the farm. Three days later, cattle on the farm began to die. However, no direct evidence supported that the anthrax outbreak occurred in Xifeng County originated from Dalian because there was no anthrax epizootics reported in Dalian. A total of 43 domestic animals died in this outbreak. These livestock were identified as animal anthrax by the local veterinarians. At least 17 dead animals were butchered and skinned by farmers and the meat or bones were distributed to local villagers. By the end of the outbreak, the majority of remnant raw meat was recalled and destroyed by incineration.

In the investigation, we found that there were 18 villagers involved in butchering sick animals and 9 of them got infected, 27 villagers involved contacting raw meat instead of butchering sick animals and one of them got infected. Chi-square analysis indicated significant differences in attack rate between the two group villagers involved butchering sick animals and contacting raw meat (χ^2^ = 10.85, ν = 1, p<0.005). So the primary risk factor was butchering infected animals. In many rural area of China, the sick or dead animals were frequently slaughtered and the meat was usually consumed by the villagers or even sold in the market to reduce economic losses. So strengthening public health education and prohibiting the sick or dead animal sale in the market are the primary measures to reduce the human anthrax in China.

### Genetic characteristics analysis of *B*.*anthracis* in Liaoning

**Fig 2:**
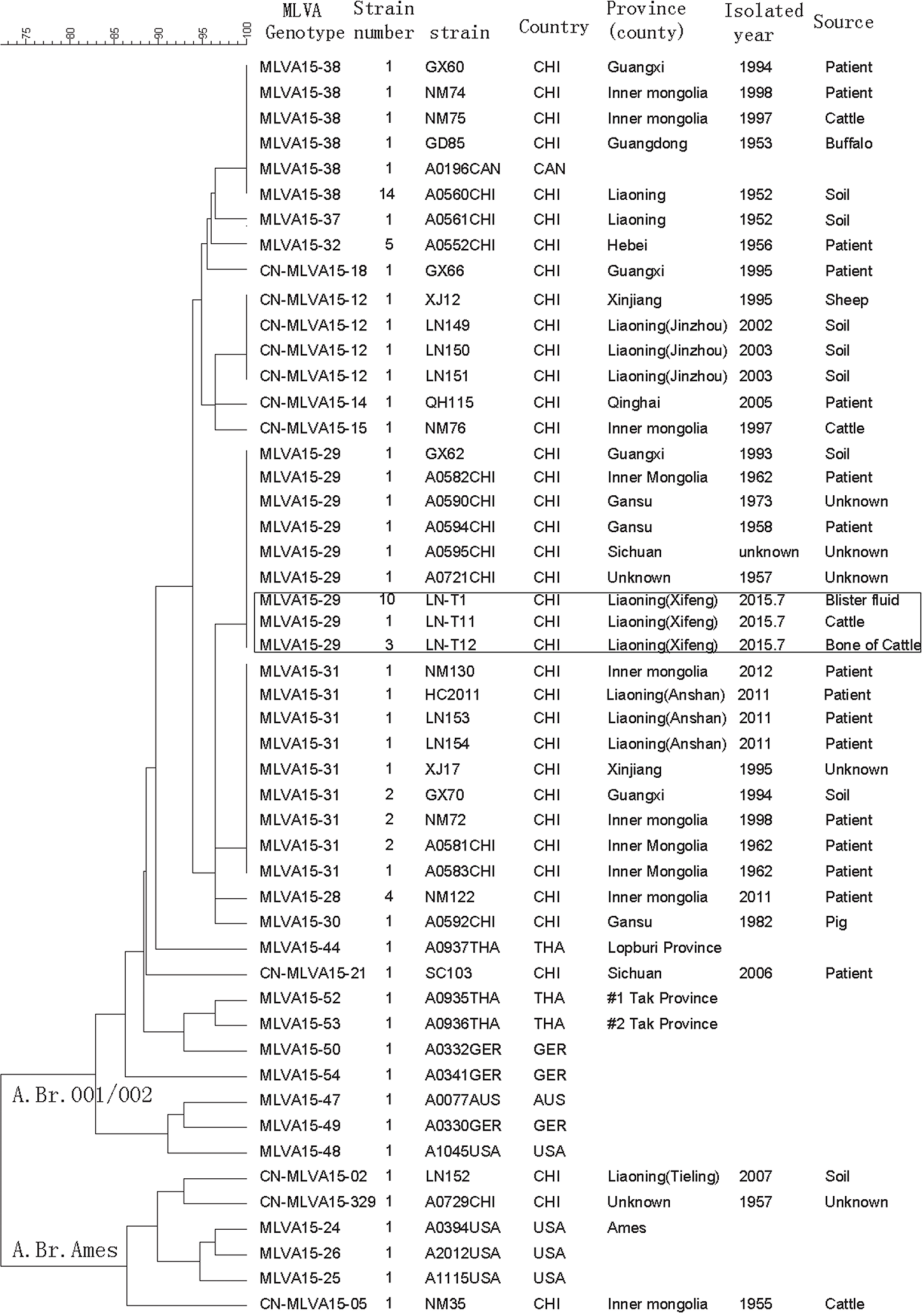
Dendrogram of MLVA15 among isolates collected from Liaoning Province and other regions.

According to canSNP analysis, all of strains/samples in the study were divided into 2 subgroups, A.Br.001/002 and A.Br.Ames. The outbreak-related strain and samples collected from Xifeng County in 2015 were classified to canSNP A.Br.001/002 subgroup, which is the major canSNP subgroup in Liaoning (Fig 2). There is only one strain (LN152, collected from Tieling Prefectures in 2007) belonged to A.Br.Ames subgroup. In order to identify the phylogenetic relationship of these strains/samples, the MLVA15 scheme was used to subtype them. All of Liaoning strains/samples were divided into 6 genotypes, and these strains/samples collected from Xifeng County in 2005 were classified to MLVA15-29 genotype. This result showed that the cattle and human cases were infected by same source.

The strains isolated from Guangxi (in 1993), Gansu (1958 and 1973), Inner Mongolia (1962), Sichuan (unknown isolated year) and one isolated in 1957(unknown the isolated sites) were also classified to MLVA15-29 genotype. It has been known that distant transportation of livestock can cause anthrax outbreak in the importing area, for example, in 2012, a human anthrax outbreak occurred in Lianyungang, Jiangsu Province where no anthrax cases have been reported for many years, epidemiological investigation found that the sick cattle as a source of infection were transported from Liaoning Province. Similarly, a human anthrax outbreak occurred in Haicheng and Anshan of Liaoning Province in 2011, the sick cattle were also found originated from other counties or areas (national anthrax surveillance data). However, there were no evidences showed epidemiological connection between anthrax outbreaks occurred in Liaoning and those in other provinces.

In previous study, 1,033 global *B*. *anthracis* isolates (from 42 countries) were divided into 12 canSNP subgroups or sublineages by canSNP analysis [4], with 5 different canSNP sub-lineages/sub-groups in China (A.Br.001/002, A.Br.Ames, A.Br.Aust94, A.Br.Vollum and A.Br.008/009) [4]. A.Br.001/002 subgroup is the most prevalent group in China and A.Br.Ames mainly distributed in northern China [19]. Our results provided supporting data to that conclusion.

Molecular subtyping approaches are a matter of importance for epidemiologic and forensic investigations [5, 22]. Van Ert et al. [4] divided 1033 *B*. *anthracis* isolates from 42 countries into 221 unique genotypes using the scheme of MLVA15. Previous study described the MLVA characteristics of *B*.*anthracis* collected from eight provinces in China (a total of 191 isolates were included), but the majority of isolates were collected from Xinjiang, and those isolates were all obtained before 1990[19]. In this study, the profiles of MLVA15 in previous literature [4, 17] were also included in MLVA analysis, which enlarged the data scope of isolates collection. In addition, with the help of the database in websites [23], genetic characteristics of *B*.*anthracis* could be compared worldwide. Thirty-six *B*. *anthracis* samples from Liaoning Province (8 strains in our collection, 15 strains in literatures, and 13 samples collected in Xifeng outbreak 2015) were divided into six MLVA15 genotypes. Fig 2 illustrated the dendrogram of clustered MLVA15 genotypes in Liaoning. The samples collected from Xifeng in 2015 formed one genotype, and the strains from other anthrax endemic regions in Liaoning (in 1952, 2002, 2003, 2007, 2011) were divided into different MLVA genotypes. Fig 2 also showed the phylogenetic relationship of other representative strains from other provinces in China.

### Genetic source tracing on *B*.*anthracis* outbreak in Liaoning Province, 2015

In order to further differentiate the event -related strains in the outbreak occurred in Xifeng, Liaoning, 2015, SNR analysis (CL10, CL12, CL33, and CL35) [18,22] was used to trace the source of infection among the samples collected from patients and livestock. The SNR profiles (CL10: 17, CL12: 15, CL33: 29, and CL35: 13) were indistinguishable among 10 samples collected from patients and 1 strain isolated from dead cattle, as well as 3 samples collected from bone or skin of dead cattle. This result demonstrated that the patients of these three events were infected by the same strain (S1 Table).

The canSNP with a low resolution is not adequate for investigation of an infectious source. One scheme combining canSNP and MLVA15 was used to analyze anthrax outbreaks in many countries, which facilitated the employment of genetic population structure comparison [14, 15, 16]. The SNR with higher resolution can differentiate strains with same MLVA15 genotype, and is used to trace the source of outbreaks [24]. In this study, MLVA method could differentiate the isolates collected in 2015 from previous isolates. SNR marker could further identified whether these patient and animal cases were originated from one infectious source.

In the study, we have reported the anthrax outbreaks that occurred in Liaoning in the recent two decades, especially an anthrax outbreak occurred in 2015, and illustrated the genetic population of *B*.*anthracis* isolated in Liaoning by means of canSNP, MLVA15 and SNR methods. Through the epidemiological investigation we found that all of the anthrax cases were caused by butchering and contacting sick animal, so strengthening public health education in China is the primary measure to prevent and control anthrax. Our result showed that canSNP with a low resolution is not adequate for investigation of an infectious source, MLVA15 and SNR could be used to track the source of the outbreak, and combination of these methods is helpful for us to judge the relationship among the cases. With the development of next generation sequencing, genome-based analysis becomes a powerful tool for the investigation of unexpected outbreaks of infectious disease.

## Supporting Information

S1 Table The profiles of SNR (CL10, CL12, CL33, CL35) related to isolates in Liaoning. (XLS)

S2 Table The profiles of MLVA15 involved in this study. The nomenclature of genotypes of MLVA15 according to Keim Genetics Lab ID Designation. For the new genotypes, the nomenclatures labeled “CN” were organized in this study.
(XLS)
